# Regional Variations in the Cellular, Biochemical, and Biomechanical Characteristics of Rabbit Annulus Fibrosus

**DOI:** 10.1371/journal.pone.0091799

**Published:** 2014-03-12

**Authors:** Jun Li, Chen Liu, Qianping Guo, Huilin Yang, Bin Li

**Affiliations:** 1 Department of Orthopaedics, The First Affiliated Hospital of Soochow University, Suzhou, Jiangsu, China; 2 Orthopedic Institute, Soochow University, Suzhou, Jiangsu, China; Van Andel Institute, United States of America

## Abstract

Tissue engineering of annulus fibrosus (AF), the essential load-bearing disc component, remains challenging due to the intrinsic heterogeneity of AF tissue. In order to provide a set of characterization data of AF tissue, which serve as the benchmark for constructing tissue engineered AF, we analyzed tissues and cells from various radial zones of AF, i.e., inner AF (iAF), middle AF (mAF), and outer AF (oAF), using a rabbit model. We found that a radial gradient in the cellular, biochemical, and biomechanical characteristics of rabbit AF existed. Specifically, the iAF cells (iAFCs) had the highest expression of collagen-II and aggrecan genes, while oAF cells (oAFCs) had the highest collagen-I gene expression. The contents of DNA, total collagen and collagen-I sequentially increased from iAF, mAF to oAF, while glycosaminoglycan (GAG) and collagen-II levels decreased. The cell traction forces of primary AFCs gradually decreased from iAFCs, mAFCs to oAFCs, being 336.6±155.3, 199.0±158.8, and 123.8±76.1 Pa, respectively. The storage moduli of iAF, mAF, and oAF were 0.032±0.002, 2.121±0.656, and 4.130±0.159 MPa, respectively. These measurements have established a set of reference data for functional evaluation of the efficacy of AF tissue engineering strategies using a convenient and cost-effective rabbit model, the findings of which may be further translated to human research.

## Introduction

Disc degeneration disease (DDD), the major cause of low back pain, has become a serious health problem and significantly contributes to healthcare expenditures [1]. Current conservative or surgical treatments for DDD can hardly reverse the biological function of degenerated intervertebral disc (IVD) cells and tissue, and may even lead to degenerative changes in adjacent vertebrae, let alone the high post-surgery recurrence rate [2], [3]. Instead, tissue engineering has emerged as a promising approach for DDD therapy by using engineered disc replacements [4], [5]. However, despite the considerable progress in engineering the nucleus pulposus (NP) of IVD, none has led to translation to clinical implementation. One of the reasons is lack of effective strategies to repair damaged annulus fibrosus (AF) [4], [6]. As a component which plays a critical role in the biomechanical properties of IVD, the structural integrity of AF is essential to confining NP and maintaining physiological intradiscal pressure upon loading [4]. Injuries of AF tissue, small or large, can lead to substantial deterioration of whole IVD which characterizes DDD [7]. Therefore, repairing/regenerating AF is essential in order to achieve effective disc repair/regeneration [8].

Nonetheless, AF tissue engineering has remained challenging because of the remarkable complexity of AF tissue [9]–[11]. Unlike NP and cartilage end plate (CEP), AF is an intrinsically heterogeneous tissue which consists of a series of oriented concentric layers surrounding NP. The biological, biochemical, and biomechanical characteristics significantly vary along its radial direction. An ideal tissue engineered AF, therefore, should recapitulate the biochemical, microstructural, and cellular characteristics of native AF tissue. This requires systematic comprehension of the regional variations of AF on both qualitative and quantitative basis, which provides well-defined native cellular and tissue benchmarks for evaluating the functional equivalence of engineered tissues [12]. Unfortunately, except for human, there are limited characterization data for AFs of other mammals. For example, rabbit is a commonly used model for IVD research taking advantage of its moderate size, ease of surgery and post-surgery analyses [13], [14]. However, lacking information of the regional difference of rabbit AF tissue has been an obstacle for appropriate construction of engineered AF.

To this end, we characterized the cellular, biochemical, and biomechanical specifics of different regions of rabbit AF tissue in this study. We isolated cells from various AF regions along its radial direction, i.e., inner, middle, and outer AF, to perform measurements at cellular level. We then performed histological and biochemical analyses of each region. Importantly, we, for the first time, employed a novel cell traction force microscopy (CTFM) technique to measure the cell traction forces (CTFs) of individual AF cells, which may serve as useful biophysical markers for characterizing AF cells from various regions. In addition to tensile test through traditional macro-scale approach, we also determined the region-wise biomechanical properties of AF tissues using nanoindentation technique. Results from this study may provide a foundation for further comprehension of AF biology as well as fundamental benchmarks for functional evaluation of the strategies of AF tissue engineering.

## Materials and Methods

### AF Tissue Harvesting

**Figure 1:**
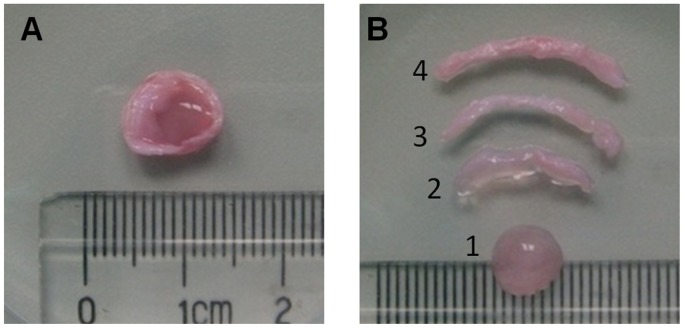
Pictures of an IVD and its sections. (A) A whole rabbit IVD. (B) With the NP (1) being removed, the AF was separated into three equal parts, i.e., iAF (2), mAF (3) and oAF (4), respectively.

The spinal column of a six-month-old New Zealand white rabbits was harvested by dissecting from the surrounding muscles under a sterile environment. The column was sectioned transversally in the middle of each disc after the muscles and ligaments were removed, and IVDs from T10 to L5 were isolated (**Fig. S1**). With the NP removed, the inner (iAF), middle (mAF), and outer AF (oAF) were carefully separated into three equal-thickness sections along its radial direction under a binocular dissection microscope. In addition, discrimination of iAF, mAF, and oAF was also possible based on the gloss appearance and the amount of hydrated matrix between the lamellae (**Fig. 1**). The animal surgery protocol was approved by the Institutional Animal Care and Use Committee (IACUC) of Soochow University.

### Isolation and Culture of AF Cells

AF tissues were minced and cells were isolated by digesting the tissue with 2 mg/ml collagenase type I (Sigma, C0130) at 37°C. After being filtered through a 200-µm filter and centrifuged, cells were re-suspended in Alpha’s modified Eagle’s medium (Hyclone, SH30265.01B) supplemented with 10% fetal bovine serum (Hyclone, SV30087.02), 100 U/ml penicillin, and 100 µg/ml streptomycin, and seeded at a density of 5×10^3^ cells/cm^2^. The cells were maintained in a humidified incubator at 37°C and 5% CO_2_. The medium was changed every 3 days.

### Cell Proliferation Analysis

The isolated primary cells from each region of AF were seeded in a 96-well plate at a density of 2×10^3^ cells per well. After predetermined periods of time, the cells were washed twice with PBS. Then 100 µl medium and 10 µl Cell Counting Kit-8 (CCK-8) (KeyGEN Biotech, KGA317) solution was added to each well. After 2 h of incubation, the absorbance at 450 nm was measured using a microplate reader (BioTek Instruments, USA).

### Gene Expression Analysis

The primary cells from each AF region were lysed and total RNA was extracted using the TRIZOL isolation system (Invitrogen). Reverse transcription was then performed using the Revert-Aid™ First-Strand cDNA Synthesis Kit (Fermentas, K1622) and oligo(dT) primers for 60 minutes at 42°C on a reverse transcription PCR system (Eastwin Life Science, Beijing). Primers for GAPDH, collagen-I, collagen-II and aggrecan were designed using the mRNA sequences deposited in Gene Bank (**Table 1**). Real-time quantitative PCR (RT-qPCR) was performed with a Bio-Rad CFX96™ Real-Time System using the SsoFast™ EvaGreen Supermix Kit (Bio-Rad). The copy numbers were normalized to GAPDH, and the fold differences were calculated using △△Ct method by referencing to the gene expression of oAFCs.

**Table 1:** Sequences of primers for RT-PCR.

Gene	Sequence	Accession number
Collagen-I	Forward: 5′-CTGACTGGAAGAGCGGAGAGTAC-3′	AY633663
	Reverse: 5′-CCATGTCGCAGAAGACCTTGA-3′	
Collagen-II	Forward: 5′-AGCCACCCTCGGACTCT-3′	NM_001195671
	Reverse: 5′-TTTCCTGCCTCTGCCTG-3′	
Aggrecan	Forward: 5′-ATGGCTTCCACCAGTGCG-3′	XM_002723376
	Reverse: 5′-CGGATGCCGTAGGTTCTCA-3′	
GAPDH	Forward: 5′-ACTTTGTGAAGCTCATTTCCTGGTA-3′	NM_001082253
	Reverse: 5′-GTGGTTTGAGGGCTCTTACTCCTT-3′	

### Histological Analysis

Freshly harvested specimens were rinsed in PBS and placed in a formalin solution for 48 h. After being rinsed with distilled water for 24 h, the specimens were decalcified for 5 days, followed by sequential dehydration in 70%, 95% and 100% alcohol and xylene, respectively. After being embedded in paraffin, the specimens were cut into 5 µm slices and mounted on slides. They were then dipped in xylene to remove the paraffin, and rinsed with alcohol of gradual concentrations. For *hematoxylin & eosin (H&E) staining*, the specimens were rinsed in water for 4 min, dipped in hematoxylin for 6 min, and rinsed with water for 5 min. Then they were placed in eosin for 1 min. *Safranin Orange - Fast Green staining* followed a similar pattern. Slides were cleaned with xylene and alcohol and then rinsed with water, after which they were dipped in 0.02% Fast Green for 3 min, 1% acetic acid for 15 sec, and finally 0.1% aqueous Safranin Orange for 3 min. Stained slides were viewed with a Zeiss Axiovert 200 inverted phase contrast microscope (Carl Zeiss Inc., Thornwood, NY) and images were recorded using an AxioVision software.

### Immunohistochemistry

After paraffin was removed, the tissue sections were rinsed with water for 2 min and then incubated in 1% hydrogen peroxide in methanol for 30 min, followed by washing with Tris buffer. Then they were incubated in 10 mM citrate buffer at 60°C for 10 min and then 2% goat serum (Gibco) for 10 min. Monoclonal antibodies to collagen-I (1∶200) (Abcam, 90395) and collagen-II (1∶200) (Millipore, II-4C11) were used. After washing with Tris, they were incubated in streptavidin peroxidase followed by 3,3′-diaminobenzidine (DAB) using an EnVision Detection Kit. Finally they were washed with water and counterstained with hematoxylin. Mouse mAb IgG1 isotype control (Cell Signaling Technology, 5415S) was used. Rabbit tendon and articular cartilage tissues were used as positive controls for collagen-I and collagen-II, respectively (**Fig. S2**).

### Biochemical Assays

Tissues from iAF, mAF, and oAF regions were weighed, homogenized, and then subjected to biochemical analyses to determine the contents of DNA, proteoglycan (PG), hydroxyproline (HYP), collagen-I and collagen-II, respectively. In brief, the DNA content was determined using quantitative fluorescence measurement of the homogenate hydrolysate mixed with bisbenzimidazole (Hoechst 33258, Sigma) [15]. The total glycosaminoglycan (GAG) content was quantified through the 1,9-dimethylmethylene blue (DMMB) dye-binding assay using a commercially available kit (Genmed Scientifics Inc, USA, GMS 19239.2) [16]. The HYP content was determined using a kit (Genmed Scientifics Inc., GMS50133.1) as described previously [17]. The contents of collagen-I and collagen-II were quantified using enzyme-linked immunosorbant assay (ELISA) kits (R&D Systems, USA). All assays were performed according to the instructions provided by the manufacturers and the absorbance was measured using a microplate reader. All measurements were normalized by the wet weight of tissues.

### Cell Traction Force Microscopy (CTFM)

The glass surface of a glass-bottomed petri dish was treated with NaOH solution (0.1 M) for 1 day and then coated with 3-aminopropyltrimethoxysilane for 5 min. The dish was then washed with deionized (DI) water, followed by incubation with 0.5% glutaraldehyde for 30 min. Next, the dish was thoroughly washed with DI water and air-dried. After that, a mixture (acrylamide, 5%; bis-acrylamide, 0.1%) was vacuumed for 20 min and thoroughly mixed with 0.2 µm fluorescent microbeads (volume ratio: 80/1), 40 µl ammonium persulfate, and 4 µl N,N,N′,N′-tetramethylethylenediamine. Eleven microliter of the mixture was then added to the center of pretreated dish and allowed to cure for 30 min at room temperature, which led to formation of a polyacrylamide gel (PAG) on the glass. Before conjugating collagen-I to PAG, its surface was first activated using N-sulfosuccinimidyl-6-[4′-azido-2′-nitrophenylamino] hexanoate (Sulfo-SANPAH, Pierce, Rockland, IL) under ultraviolet (UV) exposure. In brief, 100 µl Sulfo-SANPAH in 30 mM HEPES solution was added to the surface of PAG and exposed to UV for 5 min and then PAG was washed with PBS twice. Next, 130 µl collagen-I solution (100 µg/ml) was added to PAG surface, followed by overnight incubation at 4°C.

The collagen-coated PAG was thoroughly washed with PBS before a cell suspension containing about 3,000 cells was added to it. The primary AFCs were allowed to attach and spread on the gel for 6 h before cell images were taken. In order to acquire the images of cell and microbeads for CTF measurement, a region where individual cells resided on PAG was selected and then a phase contrast image of cells was taken. This was followed by taking a fluorescence image of microbeads, referred to as “force-loaded” image. After the culture medium was carefully extracted and the cells were removed using 2 ml 0.5% trypsin, an image of fluorescent microbeads under the same view, i.e., “null-force” image, was taken. Based on the three images, a custom-made MATLAB program was used to determine the displacement fields and compute CTFs [18].

### Nanoindentation Test

**Figure 2:**
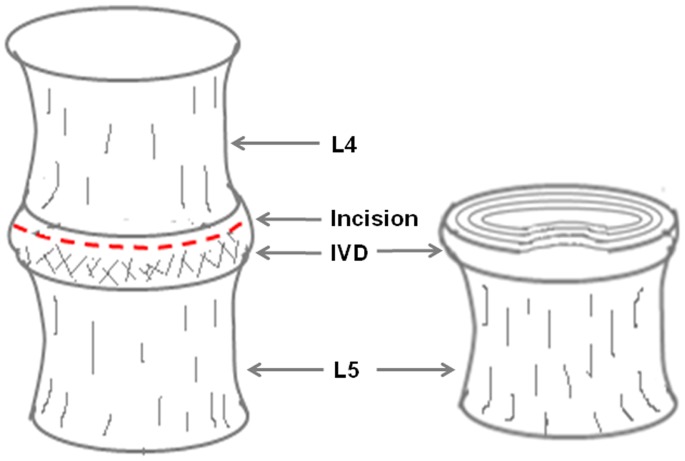
Schematic illustration of the sample preparation for nanoindentation test.

First, a motion segment including the L4 and L5 vertebrae and the IVD between them (L4–L5) was harvested from a six-month-old New Zealand white rabbit using an orthopedic saw. The L4–L5 IVD was isolated by carefully removing vertebral bone until the remaining L4 bone was approximately 1 mm thick (**Fig. 2**). Under dissection microscope and with the L5 rigidly fixed in a vice, a sharp scalpel was used to cut the IVD at a level that was 1 mm inferior to the endplate of L4 vertebrae. After being embedded with paraffin, the L5 vertebrae and L4–L5 IVD were mounted on a metal disc and then finely polished by graded sandpapers before being mounted on the nanoindentation system (**Fig. S3**). The test points were selected from inner, middle, and outer regions of AF using an optical microscope attached to the system. In order to measure the complex modulus of AF tissue, a dynamic nanoindentation protocol for viscoelastic solids was applied as previously described [19], [20]. Briefly, dynamic indentations with five running frequencies (10, 5.62, 3.16, 1.78 and 1 Hz) and 50 nm oscillation amplitude were performed using the “G-Series XP CSM flat punch complex modulus” module of the NanoSuite method on a nanoindentation facility (Agilent, Nano Indenter G200) at the axial direction using a flat indenter (diameter, 215 µm) at approximately 22°C. The samples were irrigated with PBS during test to avoid dehydration. For each AF region, at least ten points were measured.

### Tensile Test

After a whole AF was separated into three sections, i.e., iAF, mAF, and oAF, they were carefully tailored to achieve the same size for testing (**Fig. S4A**). With both ends being fixed, a sample was clamped by two fixtures (**Fig. S4B**). Tensile test was performed on an Instron 3365 testing system using a load cell of 10 N at a displacement rate of 1.5 mm/min. A validated and constant load was applied on the sample until its breakage (**Fig. S4C**). At least ten samples from each AF region were measured.

### Statistical Analysis

All data are represented as mean±SD. Statistical analyses were performed using SPSS software. Analyses of gene expression, biochemical assay, tensile test, and CTFM results were performed using Kruskal-Wallis test. Nanoindentation results were analyzed using multivariate analysis of variance (MANOVA). Difference between groups is considered statistically significant if *p* is less than 0.05.

## Results

### AF Cells

**Figure 3:**
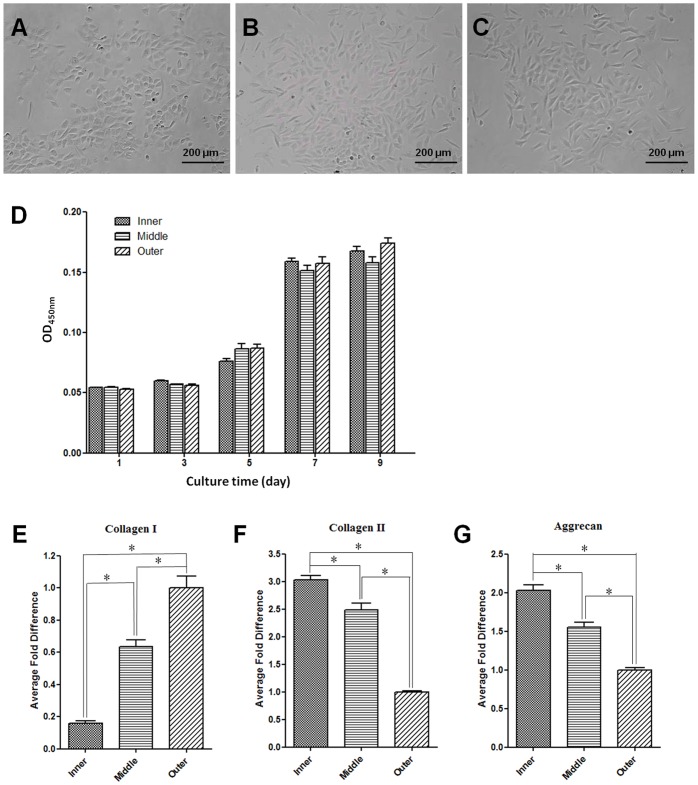
Morphology, proliferation, and gene expression of AF cells. (A–C) Phase contrast images of primary iAFCs, mAFCs, and oAFCs. (D) Proliferation of primary AFCs. (E–G) Real time quantitative PCR analysis of collagen-I, collagen-II, and aggrecan genes, respectively, for primary AFCs of various regions. Gene expression was normalized to GAPDH expression, and fold differences were calculated using the △△Ct method by comparing to gene expression of oAFCs. All data are presented as mean±SD. Asterisk (*) indicates significant difference between groups (*p*<0.01, n = 3).

The primary cells in various AF regions showed distinctively different morphology (**Fig. 3A–C**). Cells in iAF (iAFCs) had rounded, chondrocyte-like morphology, while cells in oAF (oAFCs) predominantly displayed spindle-shaped, fibroblastic morphology. The cells in mAF region (mAFCs), on the other hand, represented a mixture of iAFCs and oAFCs. The density of cells gradually increased from iAF, mAF to oAF, being 2×10^4^, 3×10^4^, and 5×10^4^ cells per gram of tissue, respectively. However, these region-specific AFCs appeared to proliferate at similar rate during culture (**Fig. 3D**). According to RT-qPCR analysis, iAFCs exhibited the least expression of collagen-I gene, while oAFCs exhibited the greatest (**Fig. 3E**). In contrast, iAFCs exhibited the greatest expression of collagen-II and aggrecan genes, while oAFCs had the least expression of them (**Fig. 3F–G**). Not surprisingly, expression of the above genes in mAFCs was between those in iAFCs and oAFCs.

### Histological Analysis

**Figure 4:**
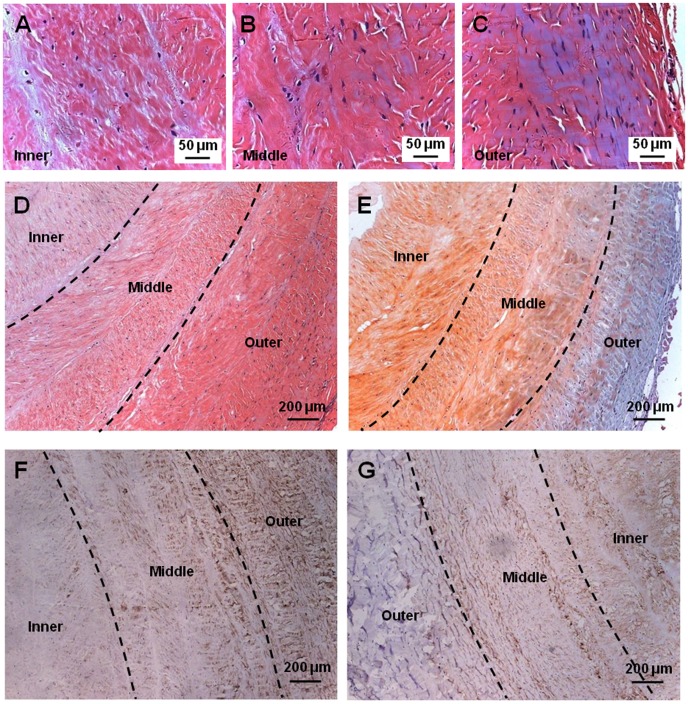
Histological and immunohistochemical analysis of AF tissue. (A–C) H&E stain of iAF, mAF and oAF, respectively. (D) H&E stain of a whole AF tissue section. (E) Safranin Orange–Fast Green stain of a whole AF tissue section. (F–G) Immunohistochemical stain for collagen-I and collagen-II expression of whole AF tissue sections.

Morphologically, the rabbit AF appeared distinctive at different zones along its radial direction. The iAF was loose and fully hydrated, while oAF was denser and less hydrated. The mAF appeared as a transition zone between iAF and oAF (**Fig. 1**). According to H&E staining, the AF tissue was mainly composed of collagen and iAF was less dense compared to mAF and oAF (**Fig. 4A–D**). The cells in iAF were round, while cells in oAF region were elongated and peripherally oriented. In addition, upon Safranin Orange and Fast Green staining iAF was intensively stained orange, indicating the existence of high PG content (**Fig. 4E**). In contrast, oAF was markedly stained with Fast Green, evidencing the presence of massive collagen fibers. It should be noted that the staining of oAF was not typical green, but bluish instead, meaning that this region was simultaneously stained with Safranin-O and Fast Green, indicative of the co-existence of PG and collagen in oAF. However, compared to PG, collagen dominated in oAF, and its content continuously decreased inward along the radial direction. Similarly, in mAF, there was gradual increase of collagen content but decrease of PG, with PG being the dominating matrix in this region. From the immunohistochemical staining of AF it was clear that there was less collagen-I in iAF compared to oAF (**Fig. 4F**). On the contrary, distribution of collagen-II showed an exactly opposite trend, i.e., more collagen-II in iAF but less in oAF (**Fig. 4G**). Again, mAF represented a transition zone between iAF and oAF, with its matrix being a mixture of collagen-I and collagen-II.

### Biochemical Analysis

**Figure 5:**
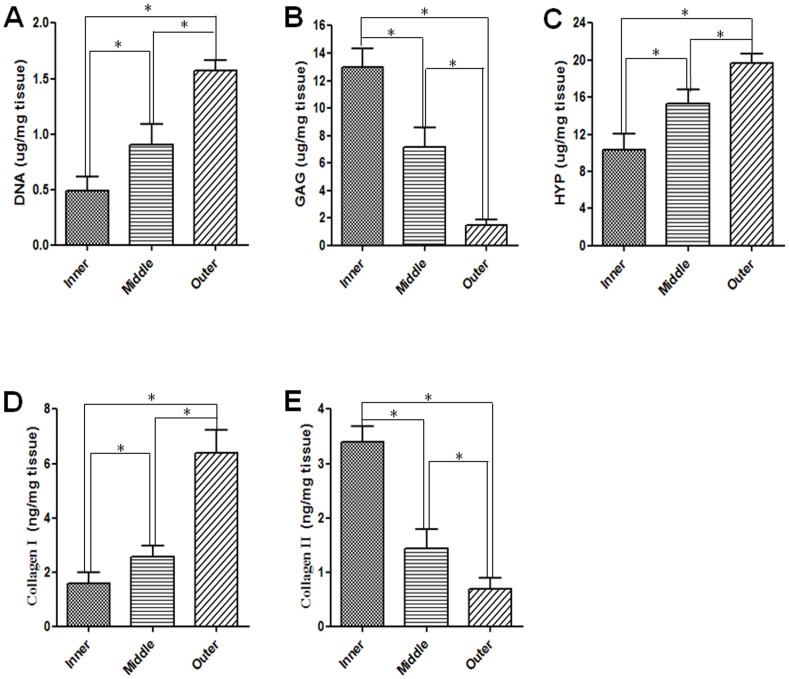
Biochemical analysis of AF tissues. (A–C) The contents of DNA, GAG, and HYP, respectively, in iAF, mAF and oAF tissues. (D–E) The contents of collagen-I and collagen-II as measured by ELISA. All measurements were normalized by wet tissue weight. All data are presented as mean±SD. Asterisk (*) indicates significant difference between groups (*p*<0.05, n≥3).

Further, the biochemical compositions of various AF regions were quantified. Clearly, the DNA content gradually increased from iAF, mAF to oAF, being 0.49±0.12, 0.90±0.18, and 1.57±0.17 µg/mg tissue, respectively (**Fig. 5A**). These measurements echoed the findings from AF cell counting and histological evaluation and confirmed that there are more cells residing in oAF than those in iAF. Meanwhile, the GAG content decreased from iAF, mAF to oAF, being 12.97±1.38, 7.19±1.39, and 1.46±0.44 µg/mg tissue, respectively (**Fig. 5B**). On the other hand, the content of total collagen protein, indicated by HYP measurement, gradually increased, being 10.37±1.72, 15.33±1.54, and 19.66±0.99 µg/mg tissue, for iAF, mAF, and oAF, respectively (**Fig. 5C**). Moreover, the content of collagen-I, as determined by ELISA, increased from 1.56±0.41, 2.56±0.42, to 6.39±0.84 µg/mg tissue, for iAF, mAF, and oAF, respectively (**Fig. 5D**). In contrast, collagen-II content decreased from 3.94±0.30, 1.43±0.36, to 0.69±0.21 µg/mg tissue from iAF, mAF to oAF (**Fig. 5E**).

### Cell Traction Force Measurement

**Figure 6:**
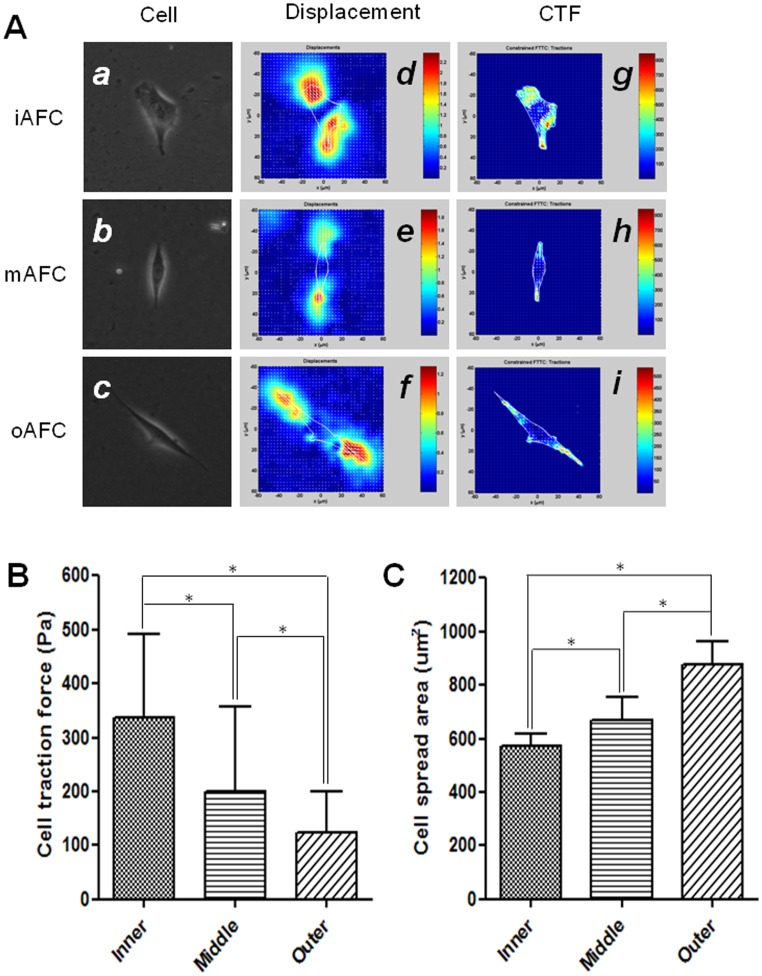
CTFM measurement of AF cells. (A) CTFM for measuring CTFs of iAFC, mAFC and oAFC. *a–c*, primary cells from each region; *d–f*, the substrate displacement fields; *g–i*, the CTF maps. (B–C) CTFs and spread areas of iAFC, mAFC and oAFC, respectively. All data are presented as mean±SD. Asterisk (*) indicates significant difference between groups (*p*<0.05, n≥30).

The cell traction forces (CTFs) of primary AFCs from different regions were measured using cell traction force microscopy (CTFM) technology (**Fig. 6A**). Apparently, the CTF gradually decreased from iAFCs, mAFCs to oAFCs, being 336.6±155.3, 199.0±158.8, and 123.8±76.1 Pa, respectively (**Fig. 6B**). However, the spread area of cells kept increasing from iAFCs, mAFCs to oAFCs (**Fig. 6C**).

### Mechanical Tests

**Figure 7:**
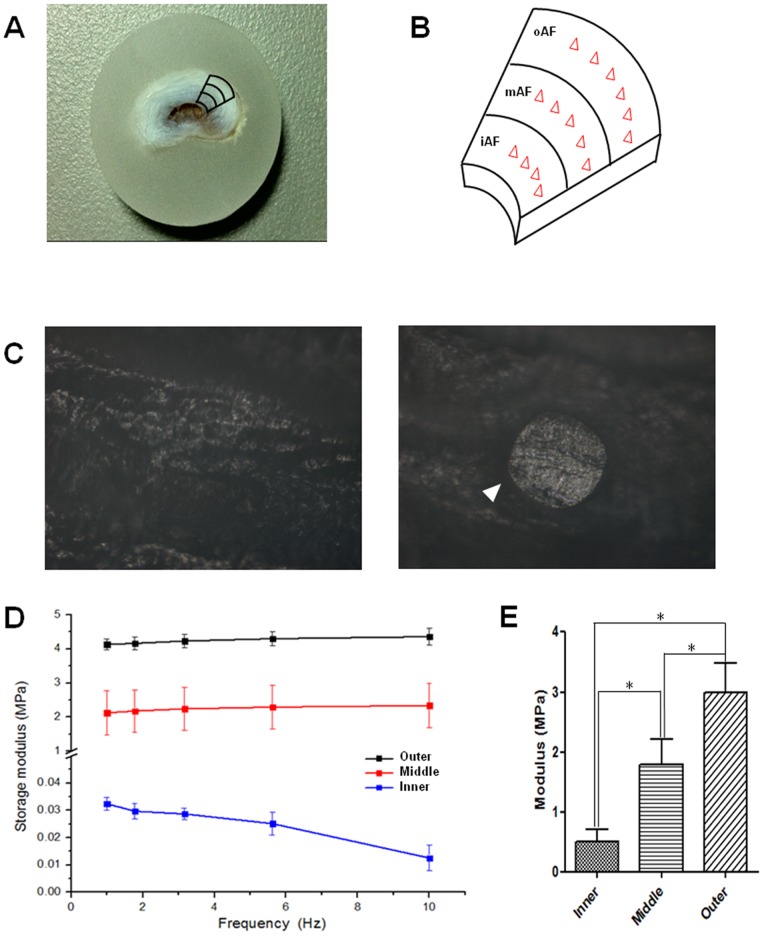
Mechanical tests of AF tissues. (A–B) The picture of a paraffin-embedded AF sample and a schematic showing how the testing regions and points were selected. Note NP was removed from the IVD. (C) The pre-test and post-test images of a sample under nanoindentation test, from which an imprint is clearly seen (shown by the arrowhead). (D) The storage moduli of oAF, mAF and iAF measured using nanoindentation at different frequencies. (E) The Young’ moduli of oAF, mAF and iAF measured using tensile test. All data are presented as mean±SD. Asterisk (*) indicates significant difference between groups (*p*<0.01, n≥10).

According to the nanoindentation results measured at different indentation frequencies, oAF and iAF consistently showed the highest and lowest storage moduli (equivalent to Young’s moduli), respectively, while mAF had moderate storage modulus in between (**Fig. 7A–D**). For instance, the storage moduli of iAF, mAF, and oAF were 0.032±0.002, 2.121±0.656, and 4.130±0.159 MPa, respectively, when measured at a frequency of 1 Hz. Interestingly, the fluctuation of storage modulus appeared to be largest for mAF compared to iAF and oAF. Such regional variations in the modulus of AF tissue were further confirmed by the results of tensile tests, according to which the Young’s moduli were 0.509±0.199, 1.790±0.328, and 2.984±0.406 MPa for iAF, mAF, and oAF, respectively (**Fig. 7E** **and Fig. S4**).

## Discussion

Despite recent exciting advancement in AF tissue engineering [21]–[25], major challenge remains toward fabricating AF replacements that are both biologically and functionally equivalent to native AF tissue. The tremendous complexity of AF at cellular, biochemical, microstructural, and biomechanical levels constitutes formidable technical hurdles [26]. In order to appropriately construct an AF alternative, such regional variations must be fully acknowledged and used as well-defined guiding benchmarks for AF tissue engineering. While there have been overwhelming studies characterizing human AF, little is known about the AF of rabbit, a very useful model for IVD studies [13], [14]. Therefore, this study set out to acquire the region-wise characteristics of rabbit AF from cellular, biochemical, structural, and mechanical aspects.

Among the various regions of AF, we found that iAF was relatively loose and highly hydrated, presumably for assisting NP to absorb axial compressive stress (**Fig. 1**) [27]. On the other hand, oAF was less hydrated and significantly more compact, which helped resist circumferential and torsion stresses. Morphologically, mAF represented a smooth transition from iAF to oAF. Histological studies revealed that iAF contained high level of PG and collagen-II, whereas oAF was fibrous and rich in collagen-I, and mAF was a mixture of its two neighbor zones (**Fig. 4**). Quantitative biochemical studies further indicated that oAF contained more DNA, total collagen, and collagen-I, while iAF contained more PG and collagen-II (**Fig. 5**). Such distinctions in the matrix composition of various AF regions were a result of the different phenotypes of cells, which produced different types of extracellular matrix (ECM) corresponding to the zone where they resided (**Fig. 3**). These observations resemble the findings of human AF studies [11], [28], [29].

While distinctions exist in the morphology and gene profiles of iAFC, mAFC, and oAFC, it is commonly agreed that there still lack specific biological markers for discriminating them. Therefore, we, for the first time, tried to characterize these cells using a novel biophysical approach, i.e., cell traction force microscopy (CTFM) (**Fig. 6**). Being the forces produced by cells and exerted on ECM, cell traction forces (CTFs) function to maintain cell shape, enable cell migration, and initiate mechanotransduction signals, and therefore play a vital role in many biological processes [30]. A close examination of CTFs of various AFCs may help better understand the cellular and molecular mechanisms of their biological roles. We founded that CTF gradually changed among AFCs of different regions (**Fig. 6B**). Interestingly, the rounded iAFCs exhibited much higher CTF than spindle-shaped oAFCs although they had much smaller size (**Fig. 6C**). This is consistent with our earlier finding and is likely related to the difference in their actin cytoskeletal structure [31]. Moreover, difference in the mechanical environment of cells in different AF regions may also count. The iAFCs were mainly subjected to high axial compressive stress and hydrostatic pressure [32], whereas oAFCs underwent peripheral tensile stresses [33]. As a result, cells from various AF regions responded with different cellular mechanical activity and in turn, different matrix synthesis and turnover of cells [34]. Nonetheless, being able to discriminate different types of AFCs, CTFM appears to be an effective technology for characterizing AFC phenotype through a simple and convenient biophysical approach.

Recently, the elasticity of ECM has proven to effectively regulate the differentiation and lineage commitment of stem cells [35], [36]. Given the fact that different types of cells from the same origin reside at various regions of AF tissue, we performed region-wise biomechanical test of AF to check whether elasticity difference existed. We employed nanoindentation technique to measure the elastic modulus of AF tissue in a belief that measurements at micro−/nano- level, a scale comparable to cell size, more accurately reflect the mechanical environment that cells indeed sense [37]. As predicted, the stiffness of iAF, mAF, and oAF sequentially increased (**Fig. 7D**), echoing the observations from previous studies using macro-scale mechanical tests [10], [38]. In addition, we also performed tensile tests for AF tissues and found the results validated the efficacy and accuracy of nanoindentation tests (**Fig. 7E**). Such a stiffness gradient of AF is correlated to the spatial distribution of its matrix components (**Figs. 3** **–** **5**) and directly associated with the mechanical loads at specific anatomical sites [10], [39]. For instance, the relatively soft iAF mainly functions to assist NP in absorbing compressive stress in axial direction. In contrast, the much tougher oAF functions to resist circumferential shearing and torsion stresses and to enable uniform hoop stress upon disc compression [10]. Therefore, instead of a simplified homogenous model, anisotropic models which take into account the biomechanical anisotropy of AF can better reflect the real situation of this tissue and should be used for AF tissue engineering [40]. In addition, the regional variation of elasticity in AF also implies that AF tissue-specific stem/progenitor cells may differentiate into various AFCs according to the stiffness of their residential region to maintain physiological turnover of matrix and repair/regenerate AF upon injury [35], [41]. It is worth noting that when the measuring frequency was increased from 1 to 10 Hz, the stiffness of both mAF and oAF slightly increased, while the stiffness of iAF remarkably decreased. Such a pattern of stiffness change of iAF resembles the pseudoplastic behavior of hydrogel, i.e., shear thinning at high frequency, again implying the NP-like nature of iAF.

In summary, the rabbit AF tissue is anisotropic and shows a typical gradient behavior of cellular, biochemical, and biomechanical characteristics along the radial direction. As a result, structurally and mechanically homogenous AF replacements are likely not able to provide the biological function and mechanical stability necessary for preventing further degeneration of other spinal components [37]. On the other hand, engineered AF tissues that recapitulate the regional variations represent a more appropriate and promising solution toward this problem. Findings from this study, therefore, have established a set of reference data for functional evaluation of the efficacy of AF tissue engineering strategies using a convenient and cost-effective rabbit model, the findings of which may be further translated to human research.

## Supporting Information

Figure S1 **Harvest of rabbit IVD tissues.** (A) A portion of spinal column from T10 through L5. (B) IVD harvesting. (C) A whole rabbit IVD.
(TIF)

Figure S2 **Positive and isotype controls for immunohistochemistry.** (A–B) Rabbit tendon tissue was stained with anti-collagen-I antibody. Positive expression of collagen-I was seen in the tissue. (C–D) Rabbit articular cartilage was stained with anti-collagen-II antibody. Positive expression of collagen-II was seen in the tissue. (E–F) Rabbit AF tissues were stained with IgG1 isotype control antibody. Negative stain was seen in the tissues.
(TIF)

Figure S3 **The setup for nanoindentation test of AF tissue.** (A) Overview of the nanoindentation test system. (B) Mounting of a paraffin-embedded AF sample on the system for nanoindentation. After the sample was mounted, its surface was checked and specific locations for indentation were identified using a microscope attached to the system. Then the optical lens was switched away and the indenter was placed for indentation test. Note the indenter was out of focus in the picture.
(TIF)

Figure S4 **Tensile test of AF tissue.** (A) A whole AF was separated into three layers, being iAF, mAF and oAF, respectively. (B) A piece of AF tissue sample was fixed for testing. (C) Elongation of AF tissue during tensile test. (D) The setup for tensile test.
(TIF)

## References

[1] Hegewald AA, Knecht S, Baumgartner D, Gerber H, Endres M, et al (2008) Regenerative treatment strategies in spinal surgery. Front Biosci 13: 1507–1525. 17981645 10.2741/2777

[2] Hakkinen A, Kiviranta I, Neva MH, Kautiainen H, Ylinen J (2007) Reoperations after first lumbar disc herniation surgery; a special interest on residives during a 5-year follow-up. BMC Musculoskelet Disord 8: 2. 17212811 10.1186/1471-2474-8-2 PMC1781070

[3] Hughes SP, Freemont AJ, Hukins DW, McGregor AH, Roberts S (2012) The pathogenesis of degeneration of the intervertebral disc and emerging therapies in the management of back pain. J Bone Joint Surg Br 94: 1298–1304. 23015552 10.1302/0301-620X.94B10.28986

[4] Hudson KD, Alimi M, Grunert P, Hartl R, Bonassar LJ (2013) Recent advances in biological therapies for disc degeneration: tissue engineering of the annulus fibrosus, nucleus pulposus and whole intervertebral discs. Curr Opin Biotechnol 24: 872–879. 23773764 10.1016/j.copbio.2013.04.012

[5] O’Halloran DM, Pandit AS (2007) Tissue-engineering approach to regenerating the intervertebral disc. Tissue Eng 13: 1927–1954. 17518718 10.1089/ten.2005.0608

[6] Adams MA, Dolan P (2012) Intervertebral disc degeneration: evidence for two distinct phenotypes. J Anat 221: 497–506. 22881295 10.1111/j.1469-7580.2012.01551.x PMC3512277

[7] Iatridis JC (2009) Tissue engineering: Function follows form. Nat Mater 8: 923–924. 19935690 10.1038/nmat2577 PMC3004472

[8] Bron JL, Helder MN, Meisel HJ, Van Royen BJ, Smit TH (2009) Repair, regenerative and supportive therapies of the annulus fibrosus: achievements and challenges. Eur Spine J 18: 301–313. 19104850 10.1007/s00586-008-0856-x PMC2899423

[9] Cortes DH, Han WM, Smith LJ, Elliott DM (2013) Mechanical properties of the extra-fibrillar matrix of human annulus fibrosus are location and age dependent. J Orthop Res 31: 1725–1732. 23818058 10.1002/jor.22430 PMC4164199

[10] Skaggs DL, Weidenbaum M, Latridis JC, Ratcliffe A, Mow VC (1994) Regional variation in tensile properties and biochemical composition of the human lumbar annulus fibrosus. Spine 19: 1310–1319. 8066509 10.1097/00007632-199406000-00002

[11] Bruehlmann SB, Rattner JB, Matyas JR, Duncan NA (2002) Regional variations in the cellular matrix of the annulus fibrosus of the intervertebral disc. J Anat 201: 159–171. 12220124 10.1046/j.1469-7580.2002.00080.x PMC1570900

[12] Nerurkar NL, Elliott DM, Mauck RL (2010) Mechanical design criteria for intervertebral disc tissue engineering. J Biomech 43: 1017–1030. 20080239 10.1016/j.jbiomech.2009.12.001 PMC2849875

[13] Masuda K, Aota Y, Muehleman C, Imai Y, Okuma M, et al (2005) A novel rabbit model of mild, reproducible disc degeneration by an anulus needle puncture: correlation between the degree of disc injury and radiological and histological appearances of disc degeneration. Spine 30: 5–14. 15626974 10.1097/01.brs.0000148152.04401.20

[14] Kroeber MW, Unglaub F, Wang H, Schmid C, Thomsen M, et al (2002) New in vivo animal model to create intervertebral disc degeneration and to investigate the effects of therapeutic strategies to stimulate disc regeneration. Spine 27: 2684–2690. 12461394 10.1097/00007632-200212010-00007

[15] Kim YJ, Sah RLY, Doong JYH, Grodzinsky AJ (1988) Fluorometric Assay of DNA in Cartilage Explants Using Hoechst-33258. Anal Biochem 174: 168–176. 2464289 10.1016/0003-2697(88)90532-5

[16] Farndale RW, Sayers CA, Barrett AJ (1982) A direct spectrophotometric microassay for sulfated glycosaminoglycans in cartilage cultures. Connect Tissue Res 9: 247–248. 6215207 10.3109/03008208209160269

[17] Stegemann H, Stalder K (1967) Determination of hydroxyproline. Clin Chim Acta 18: 267–273. 4864804 10.1016/0009-8981(67)90167-2

[18] Li B, Lin M, Tang Y, Wang B, Wang JH (2008) A novel functional assessment of the differentiation of micropatterned muscle cells. J Biomech 41: 3349–3353. 19007933 10.1016/j.jbiomech.2008.09.025 PMC2650227

[19] Chou AI, Bansal A, Miller GJ, Nicoll SB (2006) The effect of serial monolayer passaging on the collagen expression profile of outer and inner anulus fibrosus cells. Spine 31: 1875–1881. 16924203 10.1097/01.brs.0000229222.98051.9a

[20] Herbert EG, Oliver WC, Pharr GM (2008) Nanoindentation and the dynamic characterization of viscoelastic solids. Journal of Physics D-Applied Physics 41.

[21] Driscoll TP, Nakasone RH, Szczesny SE, Elliott DM, Mauck RL (2013) Biaxial mechanics and inter-lamellar shearing of stem-cell seeded electrospun angle-ply laminates for annulus fibrosus tissue engineering. J Orthop Res 31: 864–870. 23335319 10.1002/jor.22312

[22] Koepsell L, Remund T, Bao J, Neufeld D, Fong H, et al (2011) Tissue engineering of annulus fibrosus using electrospun fibrous scaffolds with aligned polycaprolactone fibers. J Biomed Mater Res A 99: 564–575. 21936046 10.1002/jbm.a.33216

[23] Bowles RD, Williams RM, Zipfel WR, Bonassar LJ (2010) Self-assembly of aligned tissue-engineered annulus fibrosus and intervertebral disc composite via collagen gel contraction. Tissue Eng Part A 16: 1339–1348. 19905878 10.1089/ten.tea.2009.0442 PMC2952129

[24] Wan Y, Feng G, Shen FH, Laurencin CT, Li X (2008) Biphasic scaffold for annulus fibrosus tissue regeneration. Biomaterials 29: 643–652. 17997480 10.1016/j.biomaterials.2007.10.031

[25] Nerurkar NL, Baker BM, Sen S, Wible EE, Elliott DM, et al (2009) Nanofibrous biologic laminates replicate the form and function of the annulus fibrosus. Nat Mater 8: 986–992. 19855383 10.1038/nmat2558 PMC3415301

[26] Nerurkar NL, Sen S, Huang AH, Elliott DM, Mauck RL (2010) Engineered disc-like angle-ply structures for intervertebral disc replacement. Spine 35: 867–873. 20354467 10.1097/BRS.0b013e3181d74414 PMC3421837

[27] Yu J, Tirlapur U, Fairbank J, Handford P, Roberts S, et al (2007) Microfibrils, elastin fibres and collagen fibres in the human intervertebral disc and bovine tail disc. J Anat 210: 460–471. 17428205 10.1111/j.1469-7580.2007.00707.x PMC2100288

[28] Horner HA, Roberts S, Bielby RC, Menage J, Evans H, et al (2002) Cells from different regions of the intervertebral disc: effect of culture system on matrix expression and cell phenotype. Spine 27: 1018–1028. 12004167 10.1097/00007632-200205150-00004

[29] Smith LJ, Fazzalari NL (2006) Regional variations in the density and arrangement of elastic fibres in the anulus fibrosus of the human lumbar disc. J Anat 209: 359–367. 16928204 10.1111/j.1469-7580.2006.00610.x PMC2100325

[30] Li B, Wang JH (2010) Application of sensing techniques to cellular force measurement. Sensors 10: 9948–9962. 22163449 10.3390/s101109948 PMC3231038

[31] Li F, Li B, Wang QM, Wang JH (2008) Cell shape regulates collagen type I expression in human tendon fibroblasts. Cell Motil Cytoskeleton 65: 332–341. 18240273 10.1002/cm.20263

[32] McNally DS, Adams MA (1992) Internal intervertebral disc mechanics as revealed by stress profilometry. Spine 17: 66–73. 1536017 10.1097/00007632-199201000-00011

[33] Stokes IA (1987) Surface strain on human intervertebral discs. J Orthop Res 5: 348–355. 3625358 10.1002/jor.1100050306

[34] Hutton WC, Elmer WA, Boden SD (1999) The effect of hydrostatic pressure on intervertebral disc metabolism. Spine 24: 1507–1015. 10457568 10.1097/00007632-199908010-00002

[35] Engler AJ, Sen S, Sweeney HL, Discher DE (2006) Matrix elasticity directs stem cell lineage specification. Cell 126: 677–689. 16923388 10.1016/j.cell.2006.06.044

[36] Discher DE, Janmey P, Wang YL (2005) Tissue cells feel and respond to the stiffness of their substrate. Science 310: 1139–1143. 16293750 10.1126/science.1116995

[37] Lewis NT, Hussain MA, Mao JJ (2008) Investigation of nano-mechanical properties of annulus fibrosus using atomic force microscopy. Micron 39: 1008–1019. 17977735 10.1016/j.micron.2007.08.009 PMC2615464

[38] Adams MA, Dolan P (2005) Spine biomechanics. J Biomech 38: 1972–1983. 15936025 10.1016/j.jbiomech.2005.03.028

[39] Buckwalter JA, Woo SL, Goldberg VM, Hadley EC, Booth F, et al (1993) Soft-tissue aging and musculoskeletal function. J Bone Joint Surg Am 75: 1533–1548. 8408143 10.2106/00004623-199310000-00015

[40] Guerin HL, Elliott DM (2007) Quantifying the contributions of structure to annulus fibrosus mechanical function using a nonlinear, anisotropic, hyperelastic model. J Orthop Res 25: 508–516. 17149747 10.1002/jor.20324

[41] Feng G, Yang X, Shang H, Marks IW, Shen FH, et al (2010) Multipotential differentiation of human anulus fibrosus cells: an in vitro study. J Bone Joint Surg Am 92: 675–685. 20194326 10.2106/JBJS.H.01672 PMC6882534

