# Supplementary figures and images for: Regional Variations in the Cellular, Biochemical, and Biomechanical Characteristics of Rabbit Annulus Fibrosus
Source: PLoS One. 2014 Mar 12;9(3):e91799. doi: 10.1371/journal.pone.0091799 (PMC3951500; doi:10.1371/journal.pone.0091799)

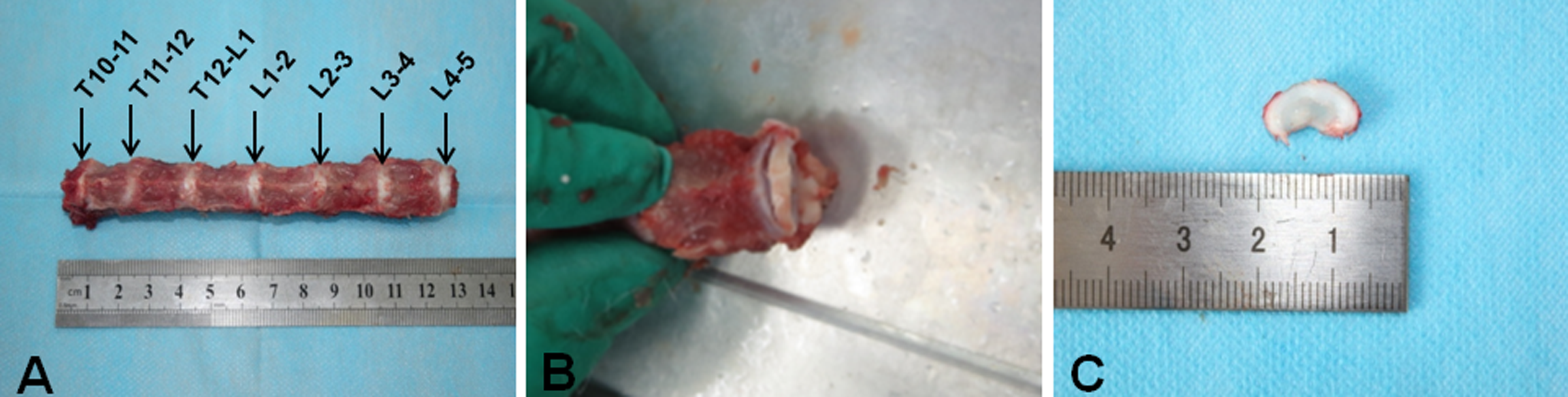

Supplement: Figure S1 — Harvest of rabbit IVD tissues. (A) A portion of spinal column from T10 through L5. (B) IVD harvesting. (C) A whole rabbit IVD. (TIF) [file pone.0091799.s001.tif]

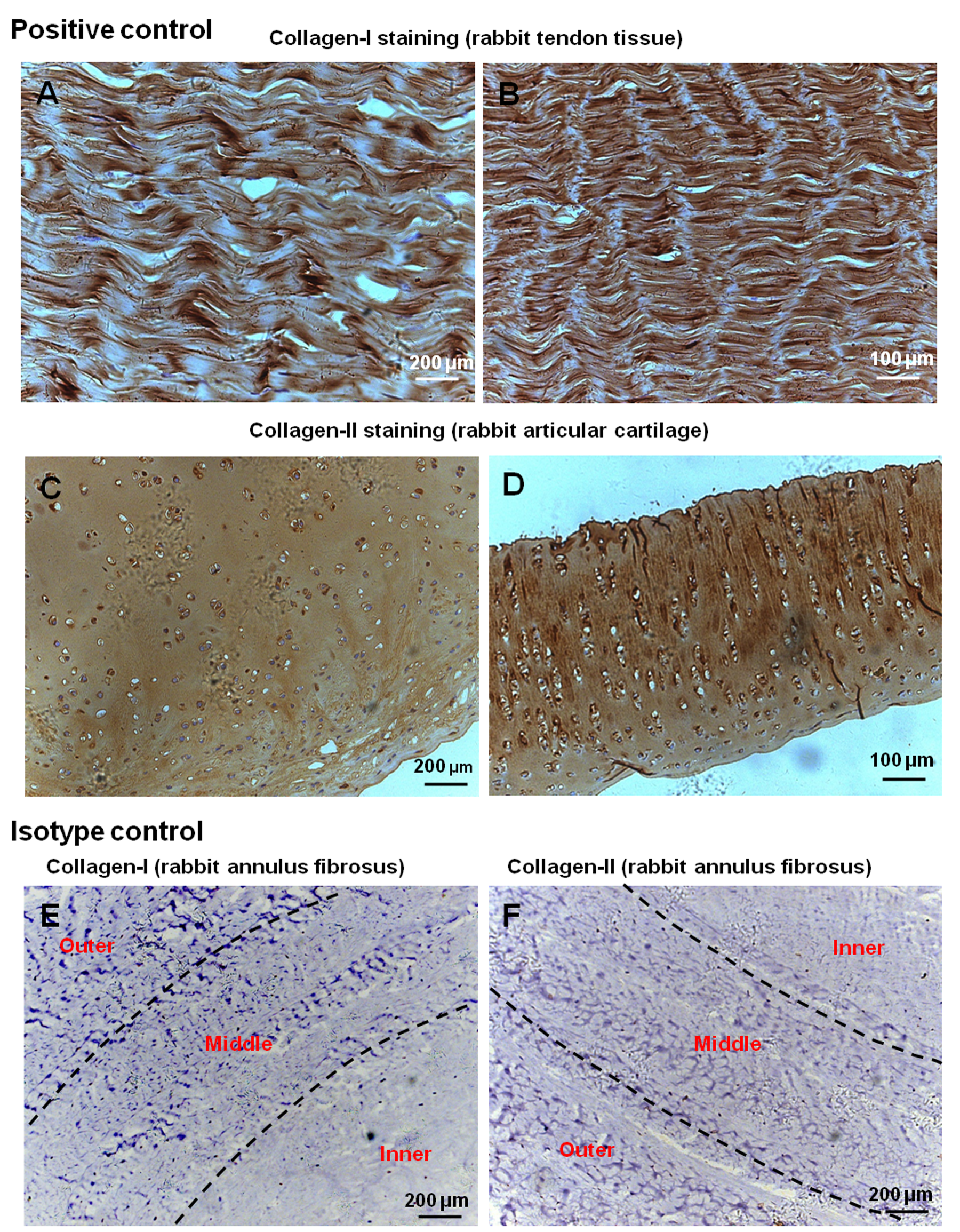

Supplement: Figure S2 — Positive and isotype controls for immunohistochemistry. (A–B) Rabbit tendon tissue was stained with anti-collagen-I antibody. Positive expression of collagen-I was seen in the tissue. (C–D) Rabbit articular cartilage was stained with anti-collagen-II antibody. Positive expression of collagen-II was seen in the tissue. (E–F) Rabbit AF tissues were stained with IgG1 isotype control antibody. Negative stain was seen in the tissues. (TIF) [file pone.0091799.s002.tif]

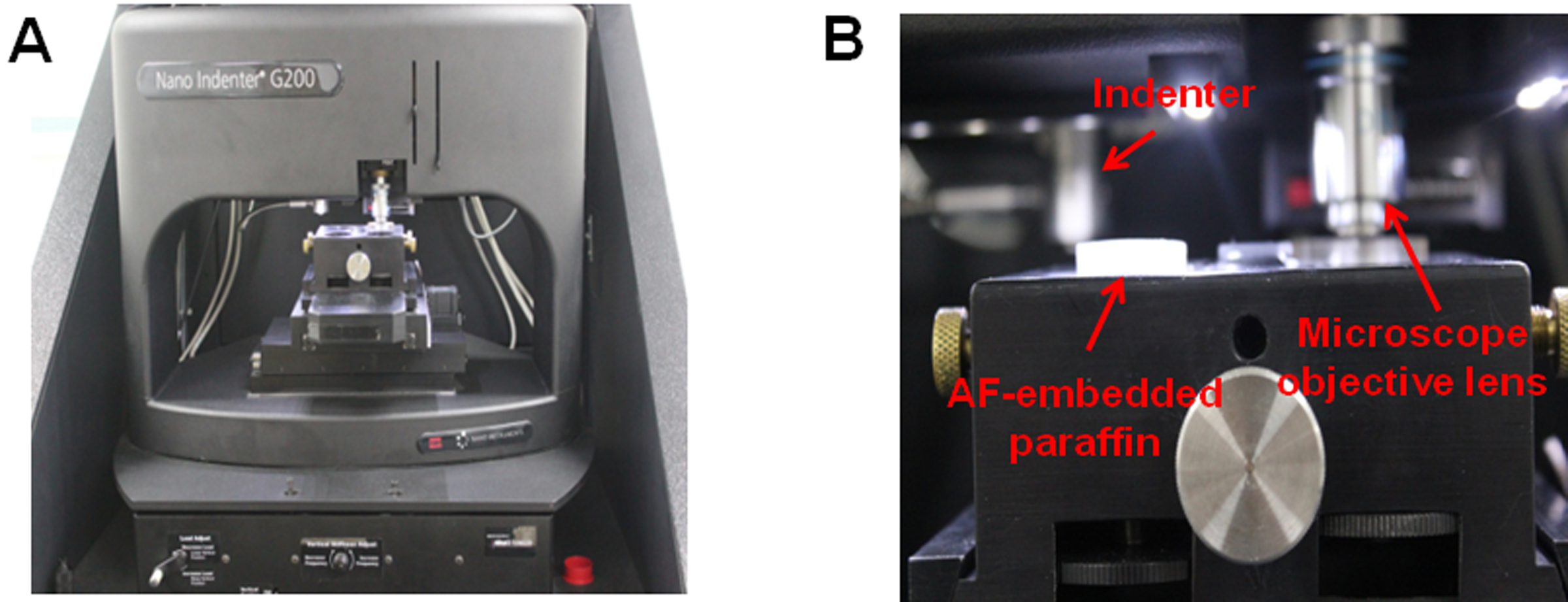

Supplement: Figure S3 — The setup for nanoindentation test of AF tissue. (A) Overview of the nanoindentation test system. (B) Mounting of a paraffin-embedded AF sample on the system for nanoindentation. After the sample was mounted, its surface was checked and specific locations for indentation were identified using a microscope attached to the system. Then the optical lens was switched away and the indenter was placed for indentation test. Note the indenter was out of focus in the picture. (TIF) [file pone.0091799.s003.tif]

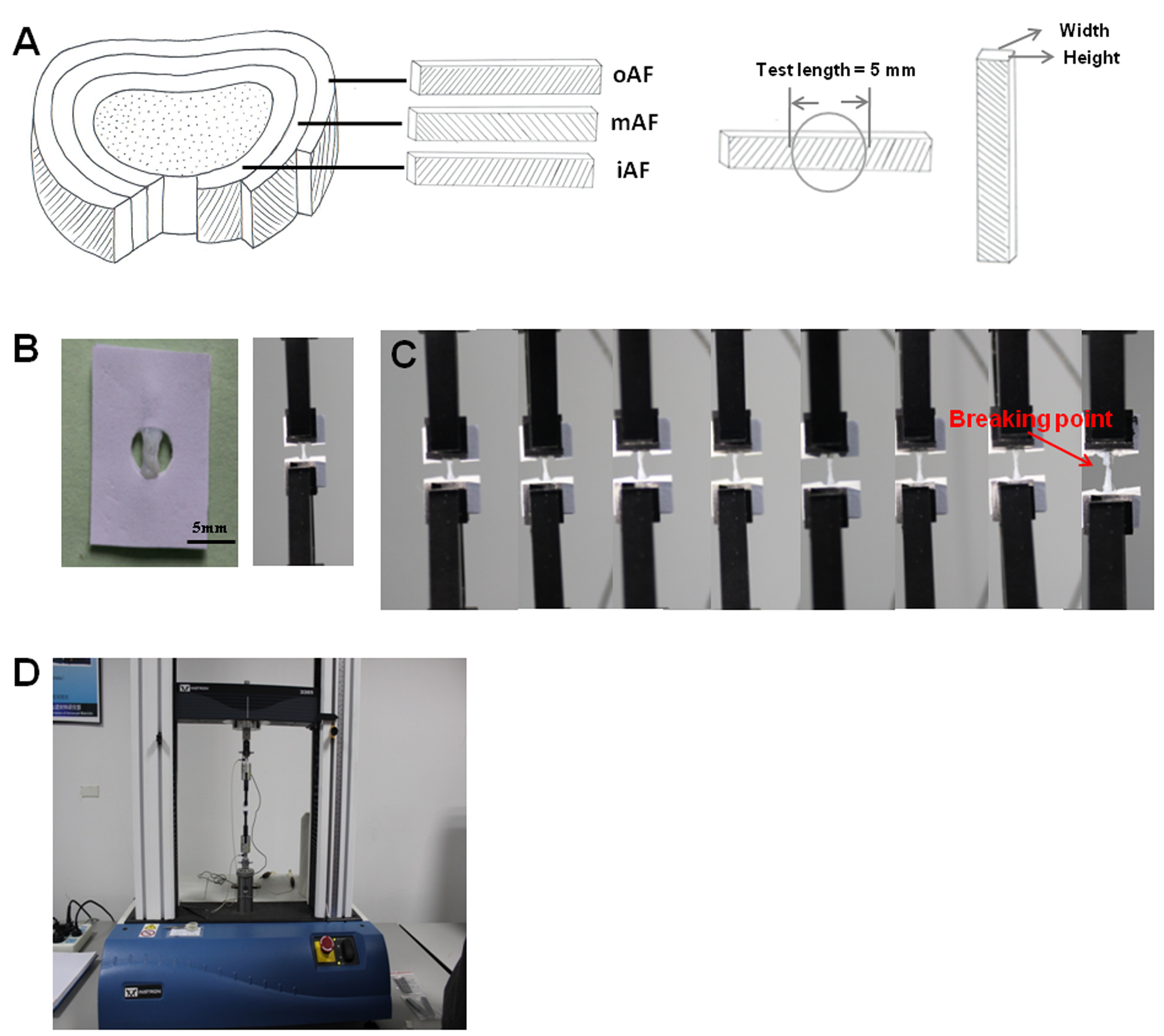

Supplement: Figure S4 — Tensile test of AF tissue. (A) A whole AF was separated into three layers, being iAF, mAF and oAF, respectively. (B) A piece of AF tissue sample was fixed for testing. (C) Elongation of AF tissue during tensile test. (D) The setup for tensile test. (TIF) [file pone.0091799.s004.tif]
